# Insomnia Symptoms in the General Population During the COVID-19 Pandemic

**DOI:** 10.3389/fpsyt.2021.762799

**Published:** 2021-11-05

**Authors:** Øyvind Halsøy, Sverre Urnes Johnson, Asle Hoffart, Omid V. Ebrahimi

**Affiliations:** ^1^Department of Psychology, University of Oslo, Oslo, Norway; ^2^Modum Bad Psychiatric Hospital, Vikersund, Norway

**Keywords:** COVID-19, sleep, social distancing, physical distancing, sleep health, epidemic

## Abstract

This empirical study aims to investigate factors associated with insomnia symptoms during the COVID-19 pandemic in 4,921 Norwegian adults. Participants were queried across two time-points, between March 31^st^ and April the 7th 2020, and between June 22nd and July 13th, 2020. Relevant risk factors and psychological correlates at the first time-point and insomnia symptoms were measured 3 months later, allowing for the investigation of concurrent associations as well as associations across time. Insomnia symptoms were measured with the Bergen Insomnia Scale. The results revealed that individuals reported higher mean levels of insomnia symptoms during the COVID-19 lockdown, compared to pre-pandemic surveys from 2008 (*p* < 0.0001, Cohen's *d* = 0.29). Individuals who predominantly socially distanced reported higher mean levels of insomnia symptoms than those who did not predominantly distance. Females, individuals with lower education levels, individuals who had lost their job, and individuals who declared having been diagnosed with an unspecified pre-existing psychiatric disorder reported the most symptoms. The regression model (R^2^ = 0.44) showed that physical exercise, was associated with less symptoms of insomnia. Symptoms of health Anxiety, symptoms of depression, unhelpful coping strategies, worry about job and economy, and older age were all associated with higher levels of insomnia symptoms. These findings highlight particularly vulnerable subgroups, as well as providing clinicians with key areas of intervention to help individuals suffering from insomnia symptoms.

## Introduction

Having to react quickly to prevent the spread of COVID-19, most countries have implemented non-pharmacological interventions (NPIs) to impede the disease until a cure could be found, or a vaccine routinely implemented (1). These NPIs encompass physical distancing procedures including major lockdowns and restriction of activity in public spheres, stay-at-home orders, closure of school and workplaces, quarantine, and isolation, as well as other social distancing protocols. Consequently, the implementation of these NPIs have involved sudden and drastic changes to the daily life of individuals (2). As the coronavirus spreads around the world, there is an increasing concern that although the NPIs are of great importance in combating the virus, the major changes to individual's everyday life stemming from isolation and social distancing measures may have immediate as well as long lasting consequences for mental health (3). Several studies suggest that the pandemic has been associated with an increase in insomnia (4, 5).

Insomnia is the most prevalent sleep disorder, affecting about 5–19% of adults globally (6, 7). However, prevalence rates vary depending on how it is measured, and which criterions are used (8). The main characteristics of insomnia are subjective complaints about difficulties falling asleep at bedtime, nighttime awakenings, early awakenings, or non-restorative sleep. These difficulties result in daytime symptoms, including fatigue, relational problems, low energy, attentional difficulties, problems with memory and mood disturbances and, irritability or general dissatisfaction (9). Insomnia carries a heavy burden on the individual afflicted (10), on society through lost work hours (11) and an increased risk of cardiovascular disease and early death (12). Insomnia is estimated to have an annual cost of 63.2 billion dollars in the United States alone (11). Previous studies highlight that insomnia is both an undertreated and an underdiagnosed disorder (9). This is echoed by Taylor (13) who suggests that insomnia has often been judged as a less severe mental health condition, however they found individuals suffering from insomnia to be 9.82 times more likely to have a diagnosis of depression and 17.35 times more likely to have a diagnosis of anxiety. Research from non-pandemic settings further suggests anxiety and depression to be risk factors for insomnia (14, 15). Insomnia symptoms also may induce range of comorbid psychiatric problems, including increases in symptoms of depression and anxiety [e.g., (13)].

Insomnia can be either episodic, recurrent, or chronic, with studies suggesting stressful events might trigger episodes (16). Previous studies further suggest that even after the stressful event has ceased, the symptoms of insomnia may persist, raising concerns that an increase in insomnia symptoms during the pandemic might persist long after physical distancing protocols are removed (16). Since insomnia itself is not only a burdensome mental illness but could potentially trigger other problems, or worsening existing conditions, understanding how it relates to the COVID-19 pandemic is of utmost importance.

The pandemic has brought with it increased uncertainty, worry and anxiety concerning one's job and economy (17, 18) and one's health (19). Increased stress, worry, anxiety and rumination might be related to insomnia by leading to hyperarousal making it harder to fall asleep (20). Within cognitive behavioral frameworks of insomnia, worry about lack of sleep is hypothesized to create a negative feedback loop, in which more worry leads to less sleep, subsequently increasing worry about not being able to fall asleep. Pillai et al. (21) found that chronicity and number of stressors increased the risk of insomnia.

Even though cognitive behavioral therapy (CBT) for insomnia has proven to be an effective treatment (22) there is still uncertainty related to which cognitive processes are involved in remission. The Self-Regulatory Executive Function (S-REF) model of mental illness (23) highlights specific mechanisms involved in the maintenance of worry and rumination which are the form of positive and negative metacognitive beliefs. Positive metacognitive beliefs are beliefs regarding the positive aspects of worry and rumination, and negative metacognitive beliefs are beliefs regarding the danger of worrying or ruminating. In the S-REF model (23), it is postulated that dysfunctional metacognitive beliefs, leads to a style of thinking called cognitive attentional syndrome. This style of thinking is characterized by unhelpful coping strategies, aimed at regulating emotions, and unpleasant thoughts, such as worry, rumination and intrusive thoughts. These unhelpful coping strategies will in turn have the unintended consequence of maintaining the symptoms rather than alleviating them. A pre-pandemic study by Palagini et al. (24) found increased levels of unhelpful coping strategies and metacognitive beliefs in individuals with insomnia symptoms, the extent to which such strategies and beliefs are associated with symptoms of insomnia in pandemic periods remains unknown.

The pandemic has brought with it a disruption of daily life in the form of stay at home orders, remote work, and lockdowns. Constructing and maintaining healthy routines are a key part of cognitive behavior therapy for insomnia, and therefore this sudden shock of increased worry and a disruption of routines might increase symptoms (22, 23).

Previous studies have found a beneficial association between physical activity and reductions in insomnia symptoms in non-pandemic periods. This association has been found for both community samples, and for individuals suffering from Insomnia (25, 26). However, this association remains uninvestigated during pandemic settings, presenting a gap in the literature.

The study will first examine the levels of insomnia symptoms following 3 months of strict physical distancing protocols and compare it to pre-pandemic estimates. Second, we will examine how these symptoms of insomnia are distributed across subgroups to identify groups that are more vulnerable to insomnia symptom. Thirdly, we will examine a multifactorial model of a wide range of relevant variables related to symptoms of insomnia. We hypothesize that the concurrent (June 22^nd^ to July 13^th^, 2020) levels of positive and negative metacognitions, unhelpful coping strategies, worry about job and economy, health anxiety, and physical activity, will predict insomnia, beyond and above, age, gender, and education.

We will then exploratively examine whether the levels of metacognitions, unhelpful coping strategies, worry about job and economy, health anxiety and physical activity at the outbreak of the pandemic (between March 31^st^ and April the 7^th^ 2020) predict the level of insomnia symptoms between June 22^nd^ and July 13^th^, 2020, beyond and above psychiatric diagnosis, age, gender, and education and the levels of the predictors at time 2 (between June 22^nd^ and July 13^th^).

## Methods

The present study is part of the Norwegian COVID-19, Mental health, and Adherence project. The pre-registered protocol of this study can be found at Clinicaltrials.gov (NCT04443361). All elements of the study are in accordance with the pre-registered protocol of the study.

### Participants and Procedure

The study was approved by the Regional Committee for Medical and Health Research Ethics (125510) and the Norwegian Centre for Research Data (802810).

The present project collected data at two time-points: The first data collection period (T1) was between March 31^st^ and April the 7^th^ 2020. The full details of this dissemination procedure are described elsewhere (27). A total of 10061 respondents participated in the study at T1. For the second wave of data collection (T2), obtained between June 22^nd^ and July 13^th^, 2020, all individuals who participated at T1 were re-invited for novel assessments. A total of 4936 individuals participated in the study at T2. Insomnia symptoms were measured only at T2. Consequently, the overall sample of the present study includes 4,936 individuals.

To be eligible for participation in the study, the respondents had to: (1) be adults, aged above or equal to 18 years old; (2) reside in Norway at the time of measurement, where all individuals experienced identical physical distancing protocols nationally; and (3) provide their consent to partake in the study.

The participants in our study were compared to a pre-pandemic assessment made by Pallesen (28) in 2008. A community sample of 5,000 people randomly drawn from the Norwegian population register. The response rate was 52.9% yielding a sample of 2,645 participants (1,292 women, and 1,352 men).

### Study Design

In this epidemiological study, the levels of insomnia symptoms in the adult population following the implementation of physical distancing protocols during the COVID-19 outbreak in Norway was investigated. It was decided to stop data collection immediately if any physical distancing protocols were changed, or if any information was given concerning the changes in these protocols during the measurement period. At both periods of data collection (i.e., T1 and T2), all physical distancing protocols remained unchanged during measurement. Additionally, no new information was provided by the government with regards to forthcoming changes in these mitigation protocols, thus eliminating expectation effects. Furthermore, the survey was administered in a random order to control for ordering effects.

### Measurement

Not all questionnaires were distributed at both T1 and T2, meaning that for some questionnaires the Cronbach's α, is only reported for one point of time.

#### Demographic Characteristics

Participants were asked about their age, sex, education, presence of pre-existing a psychiatric diagnosis, in addition to their employment status.

#### Symptoms of Insomnia

Symptoms of insomnia were measured using the Bergen Insomnia Scale (BIS) (28). The Bergen Insomnia scale (BIS) was developed to measure insomnia in accordance with the DSM-IV criteria for insomnia. The scale consists of six items, where the first four items assess difficulties with sleep initiation, sleep maintenance, early morning awakening, and non-restorative sleep. The last two items assess daytime impairments and satisfaction with sleep. Each item is given a value of 0-7, ranging from the number of days on average the person has had any troubles with the listed symptoms over the past month. The scale has a max score of 42, and a minimum of 0. The internal consistency of this scale was good in this sample with a Cronbach's α of 0.84 at T2.

#### Other Psychiatric Symptoms

Symptoms of depression were measured with the Patient Health Questionnaire (PHQ-9) (29). The internal consistency of this scale was good at T1 with a Cronbach's α of 0.89, and excellent at T2, with a Cronbach's α of 0.91. General anxiety symptoms were measured with The Generalized Anxiety Disorder Scale (GAD-7) (30). The internal consistency of this scale was excellent in this sample, with a Cronbach's α of 0.90. Health anxiety was measured using two items from the validated Health Anxiety Inventory (HAI) (31), and two questions related to the coronavirus. These questions were related to fear about being infected by the virus, and one question regarding fear of dying from the virus. The internal consistency of this scale was good at T1, with a Cronbach's α of 0.84, and was acceptable at T2 which had a Cronbach's α of 0.75.

#### Metacognitions and Emotional Strategies

The Cognitive-Attentional Syndrome Questionnaire (CAS-1) scale is a 16-item self-report scale, for measuring the severity of the Cognitive Attentional Syndrome (32). The scale consists of three subscales, unhelpful coping behaviors, positive metacognitions, and negative metacognitions. The positive metacognitions subscale had a poor internal consistency with a Cronbach's α of 0.58 at T1, and a questionable internal consistency at T2 with a Cronbach's α of 0.64. Negative metacognitions have a range of 0–100, and had good internal consistency at T1 with a Cronbach's α of 0.69 and acceptable internal consistency at T2 with a Cronbach's α *of* 0.71. Unhelpful coping strategies has a range of 0–64 and the internal consistency was good with a Cronbach's *a* of 0.89 at T1 and the internal consistency was excellent at T2 with a Cronbach's α of 0.90.

#### Adherence to Physical Distancing Protocols

Adherence to NPIs was measured by asking the participants how well they managed to follow each of the specific NPIs employed by the Norwegian government. A full list of the NPIs in place during the measurement period is provided in Supplementary Table 2 and the full list of the items measuring adherence in Supplementary Table 3, both found in the online Supplementary Material. Internal consistency was acceptable for this scale, with a Cronbach's α of 0.66. Scores range from 0 to 32, measuring the degree of adherence on a five-point Likert scale (0: No days at all; 1: Some days; 2: Half of the days; 3: Almost every day; 4: Every day), operationalized regarding the approximate number of days during which participants managed to follow each NPI during the last 14 days. Predominantly (i.e., at least 10 out of 14 days) followed distancing protocol by socially distancing from peers and public activity.

#### Physical Activity

Physical activity was measured by asking participants to what extent they had been physically active, for more than 30 min, with activities that had increased their heart rate or leading to mild palpitations during the last 2 weeks. The scale ranges from 0 to 4, ranging from 0: “Not at all,” 1: “Yes, 1 time,” 2: “Yes, 2–3 times,” 3: “Yes, 4–8 times,” or 4: “Yes, more than 8 times.”

### Statistical Analysis

To compare the level of insomnia in the sample against existing pre-pandemic epidemiological data in the Norwegian population, an independent *t*-test was utilized. ANOVAs and t-tests were used to test for differences of levels of insomnia between subgroups in the present sample. In accordance with the pre-registered protocol of this study, a hierarchical regression involving four steps was conducted. Present symptoms of insomnia (i.e., T2) was used as the criterion variable.

In the first step the influence of stable characteristics was examined, including age, gender, and education. These demographic factors further serve as control variables. In the second step concurrent levels on the (T2) state variables were included in the model, comprising positive and negative metacognitions, unhelpful coping strategies, worry about job and economy, health-anxiety and physical activity all measured. In the third step, symptoms of anxiety and depression at T2 were included in the model, to examine whether the influence of the other state variables remained when the influence of present psychiatric symptoms was controlled. In the last step, the T1 levels of positive metacognition, negative metacognitions, unhelpful coping strategies, worry about job and economy, health anxiety and physical activity, all at T1 were included. Thus, this step reveals the influence of the pandemic outbreak levels on the state variables, over and above the influence of their concurrent levels, providing information on whether these factors have a long-lasting association with insomnia symptoms when controlling for their concurrent impact and all other variables (i.e., psychiatric symptoms and demographic characteristics) in the model. Finally, the T1 levels of depression and anxiety were included to examine whether the associations of the variables at T1 remained when controlling for initial symptom levels of depression and anxiety.

Effect sizes were calculated using semi-partial correlation, to reduce the amount of bias given by confounding variables. Semi-partial correlations measure the unique contribution of each variable to the explained variance, resulting in a less biased and a more conservative estimate of correlation. Semi-partial correlation ranges from−1 to 1 and its effect sizes can be evaluated according to Cohen's criteria: criteria: small ≥0.10, medium ≥0.30, large ≥0.50. Multicollinearity and other statistical assumptions were checked. Multicollinearity was assessed with common guidelines (VIF < 5 and Tolerance > 0.20) (33). As pre-specified the threshold value for statistical significance was set to *p* < 0.01. There was no data missing in the present study as the online survey system consisted of mandatory fields of response and as no participants withdrew themselves from the study at the measurement period of criterion variable (i.e., T2).

The study relied on voluntary participation, thus being susceptible to oversampling and under sampling of certain subgroups. To address this issue, subgroups were assigned appropriate weights to match them to their known distribution in the population. Weights were assigned using the R-packages “survey” and “anesrake.” To avoid that the matching of the distribution of one factor unmatched the distribution of other variables, an iterative algorithm (i.e., raking ratio estimation). This algorithm assigns weights to factors in ordered turns, leading to a convergent set of weights for each factor that closely matches the population distribution. All slightly oversampled and under sampled subgroups (i.e., sex, age group, education, ethnic background, region, and the proportionate number of health-care professionals) were matched closely to their precise population parameters using the raking ratio algorithm. All subgroups were accurately weighted to their representative in the population, yielding an accurate and representative sample of the Norwegian adult population. The only exception was education, as the model could not converge when attempting to adjust education to population parameters. For a more thorough overview of the proportion of sampled participants, refer to Table 1. All analyses were executed in R.

**Table 1:** A table revealing the proportion of the sampled participants.

	**Sampled *N* (%)**	**Weighted *N* (%)**	**Percentage of subgroup in the Norwegian adult population**
**Sex**			
Female	3,911 (79.48%)	2,449 (49.77%)	49.77%
Male	1,010 (20.52%)	2,472 (50.23%)	50.23%
**Age group**			
18–30	1,703 (34.07%)	1,213 (24.65%)	23.20%
31–44	1,606 (32.64%)	1,270 (25.80%)	24.30%
45–64	1,344 (27.31%)	1,634 (33.20%	31.26%
65 and above	268 (5.45%)	804 (16.34%)	21.22%
**Ethnic background**			
Native	4,563 (92.27%)	4,354 (88.48%)	85.29%
Europe	274 (5.57%)	396 (8.05%)	7.58%
Asia	39 (0.79%)	117 (2.38%)	4.56%
Africa	6 (0.12%)	18 (0.37%)	1.85%
North-America and Oceania	15 (0.30%)	12 (0.24%)	0.27%
Middle- and South America	24 (0.49%)	24 (0.49%)	0.45%
**Region**			
East Norway	3,103 (63.06%)	2,862 (58.16%)	58.32%
West Norway	1,162 (23.61%)	966 (19.63%)	20.28%
Mid Norway	482 (3.54%)	826 (16.79%)	15.95%
Northern Norway	174 (3.54%)	267 (5.43%)	5.45%
**University degree**			
Yes	3,219 (65.41%)	NA	30.09%
No	1,702 (34.59%)	NA	69.91%
**Health-care professional**			
Yes	813 (16.52%)	604 (12.27%)	12.70%
No	4,108 (83.48%)	4,317 (87.73%)	87.30%

*All oversampled and undersampled subgroups were assigned appropriate weights to reflect their known distribution in the population as precisely as possible. The raking ratio algorithm converged with the following adjustments weighting the sex, age, ethnic background, and regional location of the participants, in addition to the proportion of health-care workers in the sample*.

## Results

### Demographic Characteristics

The present sample included a total of 4,921 participants. There were 2,449 (49.77%) females, and 2,472 (50.23%) men in the sample. The observed prevalence of pre-existing psychiatric disorder in the present sample was 15.75%, corresponding to the known prevalence rate in the Norwegian adult population which is between 16.7 and 25% (Norwegian Institute of Public Health, 2016).

### Symptoms of Insomnia

To test the hypothesis that the COVID-19 pandemic would be associated with an increase in symptoms of insomnia we compared the results of our survey with a sample of Norwegian adults from 2008. This sample also utilized Bergen Insomnia Scale. The mean level of insomnia symptoms in the present pandemic sample was 13.54 (*SD* = 10.16*, N* = 4,921), while the pre-pandemic sample from the same population (28) had a mean level of 10.67 (SD = 9.73, *N* = 2,645). There was a significant increase in symptoms of insomnia during the present pandemic sample as compared to the pre-pandemic sample *t*_(7, 564)_ = 11.89, *p* < 0.0001, Cohen's *d* = 0.29.

Individuals who predominantly socially distanced at T2, reported symptoms with a mean of 14.10 (*SD* = 10.23), compared to individuals who did not predominantly socially distance 11.69 (*SD* = 9.69). The difference between the groups was found to be significant, *t*_(4919)_ = −12.36, *p* < 0.001, Cohen's *d* = 0.24. Furthermore difference between current levels of insomnia and distancing at T1 was examined. Individuals who predominantly socially distanced at T1 reported a higher mean level of insomnia symptoms at T2 with a mean of 13.85 (*SD* = 10.32) compared to those who did not predominantly distance, mean 12.38 (*SD* = 9.46). The difference between the groups was statistically significant: *t*_(4919)_ = 4.19, *p* < 0.001, Cohen's *d* = 0.15. Having a psychiatric diagnosis, being younger and not having higher education were associated with significantly higher levels of insomnia symptoms. For a complete overview of the variables examined see Table 2.

**Table 2:** Differences in mean levels of insomnia symptoms, as measured by Bergen Insomnia Scale.

	***N***	**Mean (*SD*)**	***t*** **or *F***	***p***	***d***
**Symptoms of insomnia**					
**All participants**	4,921	13.54 (10.16)			
**Age group, years**			42.23	<0.001	
18–30	1,213	14.04 (8.95)			
31–44	1,270	15.51 (10.17)			
45–64	1,634	13.24 (10.88)			
65+	804	10.25 (9.48)			
**Sex**			6.59	<0.001	0.19
Female	2,449	14.49 (10.08)			
Male	2,472	12.59 (10.15)			
**Predominantly socially distanced T1**			4.19	<0.001	0.15
Yes	3,854	13.85 (10.32)			
No	1,067	12.38 (9.46)			
**Predominantly socially distanced T2**			7.08	<0.001	0.24
Yes	3,771	14.10 (10.23)			
No	1,150	11.69 (9.69)			
**Current psychiatric diagnosis**			262.00	<0.001	0.97
No	4,146	12.02 (9.36)			
Yes	775	21.61 (10.46)			
**Employment**			10.78	<0.001	0.32
Unemployed	1,502	15.86 (11.96)			
Employed	3,419	12.51 (9.06)			

### Factors Associated With Insomnia

The results of the hierarchical regression analysis for insomnia as the criterion variable are presented in Table 3. The final regression model accounted for 44% of the variance of symptoms of insomnia, adjusted *R*^2^ = 0.44. In the first step, with the stable variables: age, gender, and education the model accounted for 3% of the variance, adjusted *R*^2^ = 0.03. Higher age was associated with less symptoms of insomnia. We also found that as the education level of the participants went up, this was associated with less symptoms of insomnia. Men reported less insomnia symptoms than women. In the second step, adding state variables at T2, the model accounted for 31% of the variation, adjusted *R*^2^ = 031. Current levels (i.e., T2) of negative metacognitions, unhelpful coping strategies, worry about job and economy, and health anxiety were significantly associated insomnia symptoms, with higher levels on these variables being associated with more symptoms of insomnia. Physical activity was inversely related to symptoms of insomnia symptoms, with more physical activity being associated with less symptoms of insomnia. Positive metacognitions were not significantly related to more insomnia symptoms. In the third step, the model accounted for 44% of the variance, *R*^2^= 0.44. When controlling for concurrent levels of depression and anxiety symptoms, past symptoms of health anxiety were not related to insomnia problems. Finally, the association across time between the investigated mechanistic variables in step 2 was investigated by including these factors at T1 in the analyses. Levels of positive metacognitions and negative metacognitions both at the start of the pandemic (i.e., T1;3 months before measure of insomnia symptoms) had no significant association across time with insomnia symptoms. However, unhelpful coping strategies at the start of the pandemic (i.e., T1) had a significant association over time with symptoms of insomnia. Revealing that the more unhelpful coping strategies the individual used during the initial stages of the pandemic (i.e., T1), the greater the increases in concurrent insomnia symptoms (i.e., T2) was revealed by the individual. Physical activity during the start of the pandemic (i.e., T1) had significant associations across time with concurrent insomnia symptoms (i.e., T2). However, physical activity (T1) had a protective association across time with concurrent insomnia symptoms, with more prior physical activity at the early stages of the pandemic being associated with decreases in concurrent insomnia symptoms (T2).

**Table 3:** Results of multiple regression with Insomnia (BIS) as the dependent variable.

**Variables**	**Beta**	**SE**	***t***	***p-*** **value**	**semi Part *r***
**Step 1**.					
***R*** **^2^ = 0.03**					
Age	−0.07	0.01	−5.81	<0.001	−0.07
Gender	−1.97	0.39	−5.03	<0.001	−0.09
Education	−1.28	0.24	−5.30	<0.001	−0.11
**Step 2**.					
***R*** **^2^ = 0.31**					
Positive metacognitions T2	−0.00	0.00	−1.43	0.152	−0.01
Negative metacognitions T2	0.01	0.00	3.91	<0.001	0.05
Unhelpful coping strategies T2	0.36	0.02	17.12	<0.001	0.29
Worry about job and economy T2	0.82	0.14	5.84	<0.001	0.10
Health anxiety symptoms T2	0.39	0.11	3.38	<0.001	0.05
Physical activity T2	−0.43	0.15	−2.91	0.003	−0.05
**Step 3**.					
***R*** **^2^ = 0.44**					
Symptoms of depression T2	0.98	0.06	16.22	<0.001	0.30
Symptoms of anxiety T2	0.03	0.08	0.40	0.689	0.01
Positive metacognitions T1	0.00	0.00	0.14	0.893	0.00
Negative metacognitions T1	−0.00	0.00	−0.53	0.595	−0.02
Unhelpful coping strategies T1	0.06	0.02	2.68	<0.007	0.04
Worry about job and economy T1	−0.07	0.14	−0.53	0.600	−0.00
Symptoms of health anxiety T1	0.18	0.10	1.87	0.061	0.03
Physical activity T1	−0.48	0.15	−3.31	<0.001	−0.05

## Discussion

The present study investigated the levels of insomnia symptoms during the COVID-19 outbreak. The aim was to study the prevalence of insomnia symptoms, and the specific predictors for symptoms of insomnia. To our knowledge, this is the first study to include a measure of whether the respondents had followed the physical distancing protocols and its association with symptoms of insomnia. Comparison with a non-pandemic population sample suggest an increase in the levels of symptoms of insomnia after strict physical distancing protocols had been implemented. It also suggests that respondents who predominantly followed physical distancing protocols had higher levels of symptoms of insomnia than those who did not predominantly follow the protocols. A limitation with our design however is that we compared the mean levels of insomnia with a sample from 2008. Some studies suggest that there have been an increase in symptoms of insomnia as access to smartphones and the internet has increased (34).

### Levels of Insomnia Symptoms

Our findings, suggesting an increase in insomnia symptoms, is in line with other studies from the COVID-19 outbreak. A study with a Chinese population found about 33.7% reporting clinically significant symptoms of insomnia (12). A study from France (17) found that 19.1% of the sample reported clinically significant symptoms of insomnia. Furthermore, (35) found 37,6% of the participants in a Greek survey had clinically significant symptoms of insomnia. A recent meta-analysis found an increase in stress, anxiety and depression following the COVID-19 pandemic (36). In sum these findings point to a trend which suggests a significant increase in psychological distress and symptoms of insomnia. These studies used self-report measures, which might overestimate the prevalence rates (8).

Our findings revealed that social distancing, including activities such as quarantine and isolation, were associated with increased insomnia symptoms. Individuals who were concurrently and predominantly socially distanced reported more concurrent insomnia symptoms than their counterparts. Individuals who predominantly socially distanced at T1 – 3 months earlier –reported significantly higher levels of insomnia symptoms than individuals who did not predominantly socially distance at T1. These findings seem to preliminary suggest not only a concurrent relationship, but also an association across time between social distancing protocols and detrimental insomnia symptoms. Suggesting that these physical distancing protocols might have long term associations with sleep problems.

A possible explanation for this associations may be the disruption of daily routines and increased stress. One of the key areas of intervention for insomnia, is centered around creating stable and healthy behaviors regarding sleep (37). During the physical distancing protocols most individuals were recommended, and some were mandated to work from home. Another explanation might be stress, and previous studies have indicated that stressful events can trigger sleep problems (20). It is a matter of concern that research suggests that sleep problems can persist even after the eliciting stressful event has passed (16).

Subgroup analysis revealed an increase of insomnia symptoms in individuals who were currently unemployed. We also found that females had a higher mean of insomnia symptoms. This is in line with previous findings from the ongoing pandemic (35), and from non-pandemic samples (6) Different age groups were affected disproportionately. This is in line with previous findings from the ongoing pandemic (35, 36), but goes in the opposite direction as most of the research from non-pandemic samples (6) where symptoms of insomnia are usually increasing with older age. The groups 18–30 and 31–44 had a higher mean level of insomnia symptoms than 45–65 and 65+.

We found a significant relationship between fewer years of education, and insomnia symptoms. This finding is in line with the results of previous studies (38) and might be caused by years of education being linked to socioeconomic status. For people with lower SES the threat of losing their job, and economic recession might be increased.

The largest difference was found between individuals who had been diagnosed with a psychiatric disorder before the pandemic and those who had not, these findings converge with other findings from the ongoing pandemic (17). From non-pandemic samples, anxiety and depression have been found to be a risk factor for incident insomnia (39). Our findings suggest that the risk factors for insomnia show substantial overlap with the risk factors for anxiety and depression, meaning that these risk factors are not uniquely tied to insomnia symptoms (27).

### Factors Associated With Insomnia Symptoms

More worry about job and economy was significantly related to more levels of insomnia symptoms. Previous studies have shown that economic uncertainty and economic crisis are related to increased levels of mental health problems (40). The outbreak of COVID-19 is expected to have a negative impact on the global economy, which can lead to losses of jobs, and uncertainty about the future (2). During economic recession in Finland in the 1990's, symptoms of insomnia became more prevalent (41).

Both past and concurrent use of unhelpful coping strategies was associated with increased levels of insomnia. One of these unhelpful strategies is to drink alcohol to control emotions and thoughts. The use of alcohol to manage symptoms of insomnia has been found to be prevalent in previous studies (42). Pillai et al. (21) found that the effect of stress on insomnia is mediated by intrusive thoughts, and through coping strategies, such as thought suppression, behavioral disengagement, and substance abuse. Concurrent negative metacognition was also related to higher levels of insomnia. As specified by the metacognitive model (23), holding beliefs that worry is uncontrollable and dangerous may lead to a greater frequency of unhelpful coping strategies, and more insomnia.

Current levels of depression were also a significant predictor of symptoms of insomnia. This is in line with previous research suggesting depression to one of the largest and most consistent risk factors for insomnia (43). Taylor et al. (13) found 20% of subjects suffering from insomnia to have clinically significant levels of depression. Symptoms of current levels health anxiety was also related to more symptoms of insomnia which aligns with previous research (15). Previous symptoms of health anxiety, and current general anxiety symptoms were not significantly related to more symptoms of insomnia when controlling for all other variables. General anxiety symptoms do not contribute beyond the influence of more specific anxiety symptoms, such as worry about job and economy and worry in general. Current levels of physical activity (T2) as well as past levels of physical activity (T1) were associated with current insomnia symptoms (i.e., at T2). This finding suggests that physical activity has a negative association across time with insomnia symptoms.

### Strengths and Limitations

A major strength of the study is the elimination of sampling bias, on all our sampled variables except for education. We compared the levels of insomnia symptoms to a pre-pandemic sample from the same country, using the same measures, eliminating biases associated with different measures and populations. Another major strength of the study is the utilization of multivariate models which gives a more complex overview of the association between variables. This contrasts with univariate models, which does not control for the influence of other variables.

The study has several limitations. The cross-sectional design precludes its ability to reveal causality. Previous studies suggest a bidirectional influence of insomnia on anxiety and depression, but this is not something that we could examine in a pandemic setting. We did not have access to objective sleep measures (i.e., actigraphy or polysomnography), and previous psychiatric history of the respondents, and as a result it is impossible for us to examine the direction of this influence.

## Conclusions

Our findings suggest an increase in insomnia symptoms compared to non-pandemic periods, highlighting that the stressing nature accompanied by pandemics is associated with sleep problems. The study further revealed concurrent as well as associations across time between physical distancing protocols employed against viral mitigation and larger extent of insomnia symptoms. These findings are concerning and require further investigation into the plausible impact of protocols intruding with daily routines and sleep problems. Symptoms of depression, unhelpful coping strategies, fewer years of education and worry about job and economy found to be the most important predictors of increased symptoms of insomnia. These findings provide guidance on risk factors associated with insomnia problems during pandemic periods.

## Data Availability Statement

The datasets presented in this article are not readily available because to be able to review the dataset it has to be approved by Regional Committee for Medical and Health Research Ethics and the Norwegian Centre for Research Data. Requests to access the datasets should be directed to Øyvind Halsøy, oyvihals@math.uio.no.

## Ethics Statement

The studies involving human participants were reviewed and approved by Regional Committee for Medical and Health Research Ethics (125510) and the Norwegian Centre for Research Data (802810). The patients/participants provided their written informed consent to participate in this study.

## Author Contributions

ØH data analysis and writing. AH, SJ, and OE data collection, study design, and pre-registration. All authors contributed to the article and approved the submitted version.

## Conflict of Interest

The authors declare that the research was conducted in the absence of any commercial or financial relationships that could be construed as a potential conflict of interest.

## Publisher's Note

All claims expressed in this article are solely those of the authors and do not necessarily represent those of their affiliated organizations, or those of the publisher, the editors and the reviewers. Any product that may be evaluated in this article, or claim that may be made by its manufacturer, is not guaranteed or endorsed by the publisher.
