# Supplementary material for: Insomnia Symptoms in the General Population During the COVID-19 Pandemic
Source: Front Psychiatry. 2021 Nov 5;12:762799. doi: 10.3389/fpsyt.2021.762799 (PMC8602186; doi:10.3389/fpsyt.2021.762799)
Supplement: Supplementary file 2 [file Table_3.pdf]

Table S3. Questions measuring degree of adherence by the asking participants how well they managed to follow each of the following guidelines employed by the Norwegian government.

Item number	Question
1	I have managed to adhere to the guidelines of not leaving my home unless necessary with regards to medical visits and purchase of essential supplies.
2	I have managed to adhere to the guidelines of not traveling within the country or outside the country.
3	I have avoided social gatherings involving more than five peers.
4	I have managed to adhere to the guidelines of washing my hands regularly.
5	I have managed to adhere to the guidelines of maintaining a two-meter distance from others when I am outside my home.
6	I have managed to adhere to the guidelines of avoiding groups larger than five even when meeting peers outdoors or outside of my home/peers' home.
7	I have managed to adhere to the guidelines of keeping a two-meter distance from any peers who visit my home, with the exception of those in my household.
8	I have managed to adhere to the guidelines of avoiding all cultural gatherings and organized sports activities arranged indoors or outdoors.
